# Weight Management Effectiveness and Predictors of Dropout in Breast Cancer Survivors: A Retrospective Study

**DOI:** 10.3390/cancers15174401

**Published:** 2023-09-02

**Authors:** Edda Cava, Daniele Spadaccini, Gianluca Aimaretti, Paolo Marzullo, Beatrice Cavigiolo, Deborah Farinelli, Alessandra Gennari, Chiara Saggia, Maria Grazia Carbonelli, Sergio Riso, Flavia Prodam

**Affiliations:** 1 Unit of Dietetic and Clinical Nutrition, San Camillo-Forlanini Hospital, 00149 Rome, Italy; ecava@scamilloforlanini.rm.it (E.C.); mcarbonelli@scamilloforlanini.rm.it (M.G.C.); 2 Department of Health Sciences, University of Piemonte Orientale, Via Solaroli 17, 28100 Novara, Italy; daniele.spadaccini@uniupo.it (D.S.); gianluca.aimaretti@med.uniupo.it (G.A.); paolo.marzullo@med.uniupo.it (P.M.); 3 SCDU Endocrinology, University Hospital “Maggiore della Carità”, Department of Translational Medicine, University of Piemonte Orientale, Via Solaroli 17, 28100 Novara, Italy; 20009566@studenti.uniupo.it; 4 Unit of Dietetic and Clinical Nutrition, University Hospital “Maggiore della Carità”, 28100 Novara, Italy; deborah.farinelli@maggioreosp.novara.it (D.F.); sergio.riso@maggioreosp.novara.it (S.R.); 5 Division of Oncology, University Hospital “Maggiore della Carità”, Department of Translational Medicine, University of Eastern Piedmont, Via Solaroli 17, 28100 Novara, Italy; alessandra.gennari@uniupo.it (A.G.);

**Keywords:** breast cancer, prevention, Mediterranean diet, breast cancer survivors, weight management

## Abstract

**Simple Summary:**

Overweight and obesity are the second preventable cause of cancer, increasing the risk of its recurrence and poor outcomes, especially for breast cancer. When obesity is treated with a lifestyle intervention, breast cancer survivors may show different outcomes than the general female population, and specific causes of dropout need to be further investigated. This retrospective study aimed to investigate whether a Mediterranean diet contributed to weight management in BCS. The secondary aim was the identification of biological or anthropometrical predictors of dropout in this sample. We displayed that overweight or obese BCSs treated with a hypocaloric Mediterranean diet that concluded the 12-month follow-up period (34% of 182 women) significantly lost weight and improved their lipid profile. Moreover, lower age and higher diastolic blood pressure at baseline were found as significant predictors of dropout at 12 months. Understanding these predictors could help clinicians identify individuals who may be at a higher risk of discontinuing the intervention and design tailored strategies to support their adherence and engagement.

**Abstract:**

Background: Reducing obesity and weight gain, which often occurs during breast cancer treatment, may represent an efficient secondary or tertiary prevention against cancer. Purpose: This retrospective observational cohort study aimed to assess the impact of a Mediterranean diet on weight and anthropometric changes in women completing active breast cancer treatment. Additionally, we sought to identify factors associated with study dropout within one year. Methods: A total of 182 female patients (20 normal weight, 59 overweight, 103 obese) received personalized Mediterranean diet interventions and underwent monthly outpatient visits. Results: Dropout rates were 42.3% at 6 months and 64.1% at 12 months. Among the obese subgroup, BMI (*p* < 0.001) and fat mass (*p* < 0.05) decreased after 6 months. At 12 months, the obese subgroup showed a borderline significant further reduction in BMI (*p* = 0.062). BMI or weight loss did not predict dropout at any time point. However, age (OR = 0.91) and diastolic blood pressure (OR = 1.07) were significant predictors of dropout at 12 months. Conclusion: Implementing a Mediterranean diet can lead to weight and anthropometric improvements in breast cancer survivors. Further research is necessary to explore the long-term effects of weight loss on these individuals, identify effective dietary approaches, and consider specific predictors of dropout.

## 1. Introduction

Overweight and obesity are the second preventable cause of cancer, being related to 20% of diagnoses, and about 33% of postmenopausal breast cancer cases could be prevented by lifestyle modification [1,2]. Excessive body fat and weight gain in women, together with physical inactivity and alcohol consumption, show the strongest evidence for the risk of breast cancer (BC). White adipose tissue (WAT) expansion and hypertrophy promote hypoxia, a pro-inflammatory microenvironment with immune cell infiltration leading to cell death and cytokine imbalance. Chronic low-grade systemic inflammation, together with excessive expansion of visceral adipose tissue, contributes to insulin resistance and alters hormonal pathways, promoting tumor cell growth and metastasis [3].

Adiposity per se may not be specific enough for the risk assessment of BC, while metabolic parameters of health (e.g., insulin resistance indices such as HOMA-IR or fasting insulin) may be more biologically accurate for BC risk stratification, exhibiting a strong impact on the risk in postmenopausal women [4,5,6].

The dysfunction of visceral fat may be reversed by weight loss, improving factors contributing to the development of cancer such as insulin resistance, pro-inflammatory molecules and metabolites, circulating growth factor hormones, and adipokines [7]. Fighting obesity and weight gain, which often occurs during BC treatment, may represent an efficient secondary or tertiary prevention against cancer. At the same time, it would help in the primary prevention of chronic non-communicable diseases, including diabetes and cardiovascular disease, and would contribute to healthy aging [8]. Metabolic syndrome, associated with central adiposity and insulin resistance, is highly prevalent in breast cancer survivors (BCSs) [5,6,9], and long-term trials on weight management are needed to investigate metabolic improvements, recurrence prevention, and impact on mortality and quality of life during BC treatment. Regardless of its effect on weight loss, a recent meta-analysis has demonstrated that the Mediterranean diet (MD) can play a significant role in decreasing overall mortality among BCSs. However, there might not be a substantial difference in BC-specific mortality when comparing patients who adhere strongly to the diet with those who adhere poorly [10].

Although evidence suggests that overweight and obese BCSs have higher all-cause mortality and an increased risk of cancer recurrence with lower quality of life, there is still a lack of data to explain the impact of weight loss in this group of women and which dietary pattern would be the best approach for weight management in BCS [11,12].

In this context, it seems that the Mediterranean diet (MD) pattern could once again stand out as the most widely acknowledged and comprehensive lifestyle approach for addressing BCS. There is substantial evidence supporting its effectiveness in lowering overall mortality and minimizing cancer risk. However, it is worth noting that data pertaining to cancer recurrence remains inconclusive [13,14,15]. On a different note, when it comes to sustaining a healthy weight or attaining weight-loss goals, the Mediterranean diet has consistently demonstrated promising outcomes, even if even if the mean BMI reduction after a MD lifestyle intervention is generally not particularly high [16].

The Mediterranean dietary pattern affects several important pathways improving metabolic health and cancer risk. It (I) has a lipid-lowering effect; (II) confers protection against oxidative stress, inflammation, and platelet aggregation; (III) induces the modification of hormones and growth factors involved in the pathogenesis of cancer; (IV) inhibits nutrient sensing pathways by specific amino-acid restriction; and (V) produces gut microbiota-mediated end-metabolites [17,18]. Diet composition influences not only systemic inflammation, but also the diversity, enrichment, and composition of gut microbiota and, at the same time, can promote metabolic health, counteracting inflammation and cancer progression [19,20].

While obesity interventions report high attrition rates, the need to maximize treatment retention in BCSs with overweight or obesity requires the knowledge of factors associated with a high risk of dropout from the beginning of weight loss to maintain the efforts [21]. Existing knowledge in this field instead reports a high variability in attrition rate, with very little consistency of definitions and measurements of dropout predictors across the studies [21,22]. Moreover, no data are available concerning BCSs.

In this 24-month retrospective study, given the reduced time span preventing the examination of survival rates, our primary focus was to investigate whether, within this particular cohort of women, the implementation of a Mediterranean diet following the completion of active treatment for BC could lead to weight and anthropometric improvements. The secondary objective was to determine whether any of the examined parameters were associated with the dropout rate during the one-year study period.

## 2. Materials and Methods

### 2.1. Patients

Between June 2014 and December 2020, patients diagnosed with BC by the Oncology Department were referred and enrolled by the Dietetics and Clinical Nutrition Unit at AOU “Maggiore della Carità” in Novara, Italy, in this single-center, retrospective, observational cohort study, and were followed up for 2 years.

The study was approved by the Internal Review Board and Ethics Committee of the hospital and performed following the current legislation on observational studies and the Declaration of Helsinki to allow the retrospective utilization of the outpatient data (CE 124/2022), and to allow future contact with the subjects for further investigations.

To be eligible for the study, patients had to be: (I) free from disease (stage I or II BC), regardless of cancer type or hormone receptor status (ER, PR, HER); (II) at the end of their cancer therapy and/or surgery, and after the end of chemo and/or radiotherapy (oral hormonal therapy could be in place during the study); (III) between 30 and 80 years old. Exclusion criteria were: (I) a second cancer diagnosis in another site; (II) any acute-state disease; (III) any psychiatric disorder that could impair the ability to freely consent or comply with the study requirements and dietary therapy; (IV) high physical activity level (over 300 min/week); and (V) alcohol or drug abuse.

Dropout patients were considered those who did not show up to the main time point visits: T2 (6 months), T3 (12 months), and T4 (24 months), and the rest of the follow-up visits. If a patient missed one monthly visit but showed up in the subsequent follow-up, data were included for the nearer time point available (T2, T3, or T4), and that patient was not considered a dropout.

Patients were considered normal weight if their BMI was lower than 25 Kg/m^2^, overweight if their BMI was ≥25 Kg/m^2^ and <30 Kg/m^2^; and obese if they had BMI ≥ 30 Kg/m^2^ divided into class I (BMI ≥ 30Kg/m^2^ and <35 Kg/m^2^), class II (BMI ≥ 35 Kg/m^2^ and <40 Kg/m^2^), and class III (BMI ≥ 40 Kg/m^2^).

### 2.2. Intervention and Dietary Characteristics of the Traditional Mediterranean Diet

At baseline (T1), a physician and a dietitian conducted all the assessments and followed patients up with monthly visits. After a clinical screening and evaluation (see following), patients received a dietary intervention based on the MD pattern. For normal weight subjects, after assessing the exact measurement through a 24 h recall questionnaire, the diet was normocaloric. For overweight and obese women, the dietician formulated a hypocaloric diet aimed to obtain a target of 8–10% weight loss, applying a 500–1000 Kcal deficit from the estimated energy requirement, calculated as a mean between the 24 h recall and LARN 2014 formulas [19]. The MD was personalized and constituted 50–55% of the total daily energy (TDE) from carbohydrates, <10% of the TDE from sugars, 0.8–1.2 g/Kg/ideal body weight of proteins, and about 30% of the TDE from fats, with extra-virgin olive oil (EVOO) as the main source of fats and fish as a source of omega-3 fatty acids—eicosapentaenoic acid (EPA) and docosahexaenoic (DHA).

Qualitative characteristics of the MD were: (1) Minimally processed whole grains and legumes as the staple food; (2) a huge diversity of fresh and seasonal vegetables consumed daily and fresh fruits; (3) main fat used as condiment represented by cold-pressed extra-virgin olive oil, seldom or never by butter and cream, and occasionally fat derived from nuts and seeds; (4) moderate consumption of fish (2–3 times weekly); (5) low consumption of dairy products (mainly local cheese, milk, and yogurt); (6) red and processed meat consumed in very low frequency (only once every week or two) and amounts; (7) alcohol mainly represented by wine, consumed in low to moderate amounts (one glass per day for women and two glasses per day for men), preferably during meals [23].

If the target weight loss was reached before the end of the 2-year follow-up period, the diet was recalculated to obtain maintenance of the results, avoiding weight regain.

### 2.3. Anthropometric and Biochemical Measurements

At baseline (T1), data collected for analysis included clinical history, medications, and anthropometrics (height without shoes was measured to the nearest 0.1 cm using a Harpenden stadiometer, and body weight was measured with light clothing to the nearest 0.1 kg using an electronic scale; BMI was calculated in kg/m^2^ as body weight divided by squared height; waist circumference was measured with a non-elastic measuring tape in the mid-point between the lowest rib and the iliac crest during expiration and recorded to the nearest 0.1 cm), and blood pressure was measured by digital instrument in mmHg after participants had been sitting quietly for at least 15 min, with their right arm supported at the level of the heart and feet resting flat on the floor.

Every 1 or 2 months for a period of 24 months, a dietitian collected dietary records using a 24 h dietary recall, along with anthropometrics; medication modifications were recorded during the study, and subjects were instructed to keep physical activity at the same level during the study, to avoid any bias in the data interpretation, as its role is beneficial in cancer prevention and contributes to weight loss as well. The dietetic counselling was aimed at improving adherence to the MD.

**Figure 1:**
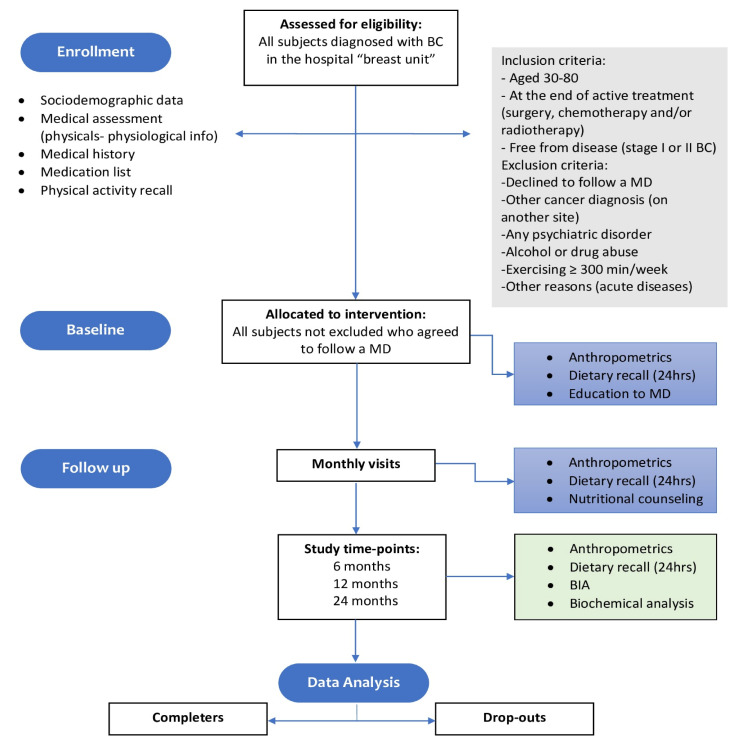
Flow diagram of the study. BC: breast cancer; MD: Mediterranean Diet; BIA: bioimpedence analysis.

In addition, at the baseline (T1) and after 6, 12, and 24 months (T2, T3, and T4), body composition assessment was performed through bioimpedance analysis to evaluate fat mass, fat-free mass, and hydration status using a BIA 101 Akern instrument, while blood samples obtained after 12 h overnight fasting were tested using standardized methods in the Hospital’s Laboratory, including the following biochemical parameters: glucose, insulin, HbA1c, lipids (total cholesterol, HDL cholesterol, and triglycerides), liver function. Insulin resistance at fasting was calculated using the formula of Homeostasis Model Assessment—Insulin Resistance HOMA-IR: [fasting insulin (μUI/mL) × fasting glucose (mg/dL)]/22.5]. The methods used in this study are summarized in a flow chart (Figure 1). 

### 2.4. Statistical Analysis

Descriptive characteristics of the sample divided into BMI subgroups were expressed as mean ± standard deviation or as median with the interquartile range depending on whether the variables had a normal or non-normal distribution. Non-normal variables were transformed into lognormal distributions accordingly. Statistical differences between subgroups were calculated with a one-way ANOVA test and Bonferroni post-hoc analysis. A Wilcoxon signed-rank test or paired samples T-test was performed for calculating statistical differences between variables at different time points.

To calculate the sample size, the main outcome considered for the analysis was the low-calorie-Mediterranean-diet-induced weight loss, considered similar to other published literature [24]. Therefore, a sample of 59 subjects was sufficient to show a reduction of 2.2 Kg above the upper limit of the normal range (ULN) with a standard deviation (SD) of 15 Kg and a 90% power, with 95% significance at *p* < 0.05.

Since the dropout rate was 85.4% at 24 months and only 26 patients showed up to this follow-up visit, we analyzed only patients with data at 1 year. After investigating whether BMI class was associated with dropout probability at 6 and 12 months with a Chi-squared test, dropout analysis was carried out by analyzing statistical differences (with an independent measures *t*-test, Mann–Whitney U test, or chi-squared test depending on the nature of the dependent variable) among baseline parameters in patients that dropped out vs completers at 6 months or at 12 months. A logistic regression model for multivariate analysis was used to identify predictors of attrition, and results were presented as odds ratios. Results were considered significant at *p* < 0.05. Variables were included in the model given their availability, importance, and clinical relevance. Statistical analysis was carried out with Statistical Packages for Social Sciences (SPSS, Chicago, IL, USA, IBM) version 25.

## 3. Results

**Figure 2:**
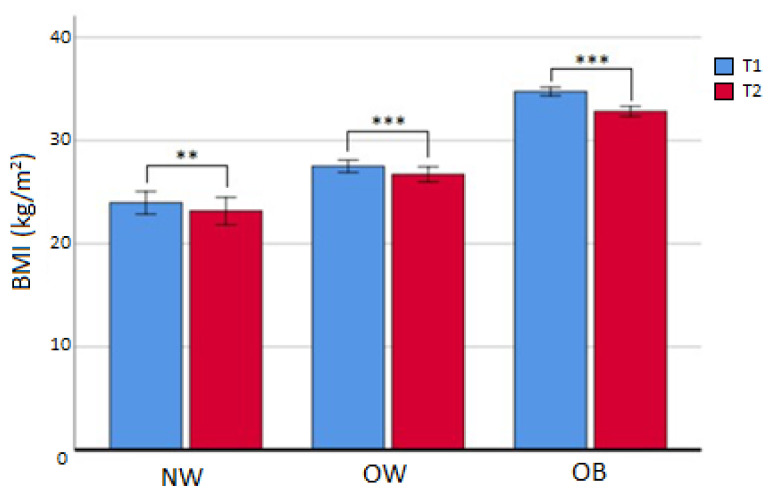
Changes in BMI in completers at 6 months. NW: normal weight; OW: overweight; OB: obese; ** = *p* < 0.01; *** = *p* < 0.001. T1 = baseline; T2 = 6 months.

**Figure 3:**
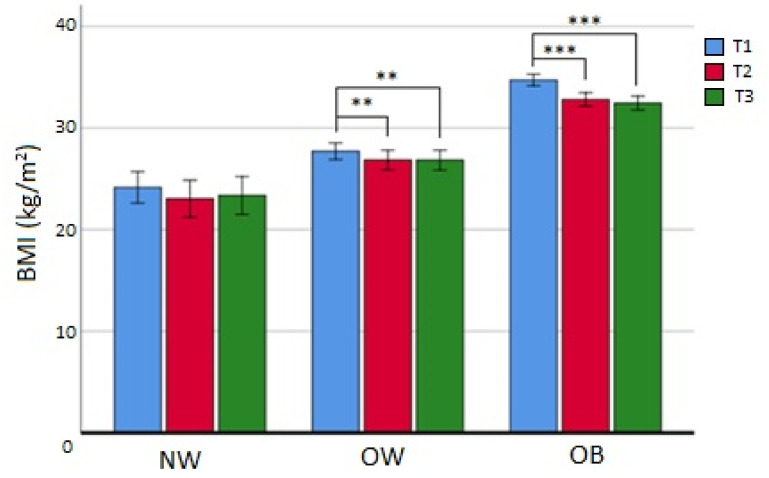
Changes in BMI in completers at 12 months; NW: normal weight; OW: overweight; OB: obese; ** = *p* < 0.01; *** = *p* < 0.001. T1 = baseline; T2 = 6 months; T3 = 12 months.

- During our study period, dietary intervention was provided to 182 female patients, out of which 20 were normal weight (NW), thus receiving a normocaloric MD, while 59 were overweight (OW) and 103 were obese (OB), thus receiving a hypocaloric MD according to their needs. In the whole sample, the mean age was 53.9 years, and the mean BMI was 31.6 Kg/m^2^. Table 1 illustrates demographic, lifestyle, and therapy characteristics of participants at the beginning of the study, while Table 2 shows relevant anthropometric and biochemical parameters, with statistical differences analyzed between BMI subgroups.

- After the first 6 months (T2), 77 subjects (42.3%) had discontinued treatment and 4 patients were deceased. Out of the 101 that continued, BMI was reduced in all subgroups (Table 3, Figure 2), in particular for OW (from median value 27.4 to median value 26.6 kg/m2; *p* < 0.001) and OB (from median value 33.5 kg/m^2^ to median value 31.8 kg/m^2^; *p* < 0.001). In the OB subgroup, a reduction in FM% compared to baseline (Δ = −3.30 ± 3.22 %; *p* < 0.05), an increase in HDL cholesterol (*p* < 0.05), and a decrease in triglycerides (*p* < 0.05) were also registered. No difference was seen in the dropout rate at 6 months between different initial BMI subgroups (χ2 = 3.099; *p* = 0.212). The prevalence of NW/OW/OB and attrition rates are displayed in Table 4. BMI was not independently linked to attrition, and after dividing the whole sample into completers and dropouts, the investigation of attrition differences showed no statistically significant results (Table 5).

- At 12 months (T3), 61 patients (33.9% of the initial sample) had continued the study, and 40 (21.9% of the initial sample and 34.2% of all dropouts) were lost at follow-up. Patients that completed T3 and were in the NW subgroup, contrarily to T2 completers, did not reach statistical significance for BMI reduction, while OW patients maintained the same BMI between T2 and T3 (*p* = ns). Finally, subjects in the OB group, even if the difference between medians was not statistically significant, appeared to have additionally reduced their BMI (from T1 median value BMI_OB_ 33.7 to T3 median value BMI_OB_ 31.9 kg/m^2^; *p* = 0.062) (Figure 3). Also in this situation, there were no observed differences in the dropout rate at 12 months among the BMI subgroups (χ^2^ = 1.120; *p* = 0.571). Nevertheless, the analysis of differences at baseline according to T3 completers and dropouts showed that age (*p* < 0.05), menopausal status (*p* < 0.01), HDL cholesterol (*p* < 0.05), and diastolic blood pressure (DBP) (*p* < 0.05) were statistically different (Table 6), while weight loss or BMI loss was not different in any BMI subgroup (*p* = ns). Finally, multivariate logistic analysis evidenced that lower age (in interaction with menopausal status) (OR_age/mp=no_ = 0.91; CI95%: 0.80–1.01; *p* = 0.092; OR_age/mp=yes_ = 0.91; CI95%: 0.82–1.0.99; *p* < 0.05) and higher DBP (OR = 1.07; CI95%: 1.02–1.12; *p* < 0.01) at baseline were significant predictors of dropout at 12 months.

- At 24 months, only 26 patients (14.6% of the initial sample) continued the study for the last visit. Only 2 patients were part of the NW group (7.7% of the whole sample and 10.5% of the NW original group), while OW and OB groups had 7 and 17 women continuing till 24 months, respectively (26.9% and 65.4% respectively of the whole sample, and 12.3% and 16.7% of their original group). Due to the high attrition, we did not analyze data from these patients as the results would be underpowered.

## 4. Discussion

Obesity is considered a risk factor for BC occurrence and worse prognosis [22,23,24], while healthy dietary patterns, such as the MD, have been associated with a decreased risk of BC, especially in postmenopausal, hormone-receptor-negative women [14,25]. Nonetheless, there is still a lack of data to explain the impact of weight loss in BCSs in terms of recurrence and survival [11].

The significance of the MD in enhancing overall survivability in BCS, as established through recent meta-analysis findings, is particularly noteworthy. However, it is important to highlight that in this meta-analysis, Chen and colleagues did not reveal a notable impact of higher MD adherence on BC-specific mortality, with the observed survivability improvement attributed to the MD’s role in averting other mortality causes [10]. 

The potential long-term combined effects of adopting an MD pattern specifically tailored for weight loss have not been thoroughly investigated. Although lacking definitive data, there is reason to speculate that this integration could further enhance the overall survival of these patients. To achieve this goal, it is crucial to ensure that BCSs maintain a high level of compliance and experience minimal dropout rates during these weight management programs.

In this context, our study demonstrates that MD in women after a breast cancer treatment is feasible, allowing a reduction in body weight, BMI, and waist circumference in the overweight and obese subgroups. On the other hand, the attrition rates reported were extremely high, with younger women being more inclined to drop out after 1 year.

While studies with larger samples have also shown the efficacy of weight-loss programs delivered in longer terms, we were able to demonstrate a significant reduction in weight, BMI, and waist circumference in the OW and OB group both for 6-month and 12-months completers [22]. We also observed a significantly reduced fat mass percentage after six months of weight management in BCSs with obesity. The implementation of bioimpedance analysis as a supplementary tool to evaluate anthropometry is important, since there is still debate about the different impacts of BMI and adiposity as independent risk factors both for BC risk and recurrence, in particular when comparing pre- and postmenopausal women [26,27].

Finally, at 6 months, improvements in HDL cholesterol and triglycerides, which are signs of metabolic benefits associated with the lifestyle treatment, were evident. These results are in line with other studies on obese BCSs [28,29].

From a clinical point of view, since we were able to demonstrate that these health benefits were significant only in the fraction of patients that completed all time points, our results are fairly dependent on the retention rate itself. Notably, at 6 months (T2) and 12 months (T3), only about 55% and 34%, respectively, of the initial sample showed up at the control visits, and we do not know how health parameters evolved in the dropout fraction.

Regardless of uncertainties in mechanistic pathways and overall compliance to the diet, oncology societies still recommend weight management to reduce body weight and fat mass as a cancer prevention tool in clinical practice [8,12]. Notably, overweight and obese states have been reported to influence treatment outcomes in terms of cancer treatment regimens (dose-cap chemotherapy, less radical surgery), differential pharmacokinetics and toxicities, and surgical peri-operative complications [30,31]. 

Concerning our secondary aim, from among the studied parameters, we did not find any statistically significant predictor of dropout at 6 months, but at 12 months, it appears that BCS were more likely to drop out if the initial age was lower and HDL cholesterol and diastolic blood pressure were higher. Despite literature reports that during weight-loss programs both initial BMI and expected 1-year BMI are associated with dropout rate [32,33,34], we were not able to find any association between initial BMI and this probability. In addition, weight loss at 6 months was not a predictor of dropout at 12 months in any of the BMI subgroups. Losing weight could be a less impactful motivating factor in BCSs while following a lifestyle intervention, since a healthy and anti-inflammatory dietary pattern may be considered by patients an independent tool for improving their health and reducing the chances of recurrence [35]. Nevertheless, BCSs can experience more physical and psychological barriers to deal with during and after the treatment [36]. Compared to men, women with a cancer history are less likely to drop from a weight management program. Still, in our female sample, we observed a similar attrition rate to than in studies on the general population [35,37,38,39].

According to our analyses, the interaction between menopausal state and age was a predictor of dropout, and this is consistent with other studies that analyzed patients without cancer following a lifestyle intervention for weight loss [32,38]. Previously, a large randomized controlled trial in BCSs showed that younger female BCSs tend to lose less weight during a weight management program than to women aged 55 or older, and this may be due to higher family obligations, work demands, as well as greater psychosocial distress after diagnosis compared to that in older patients [29]. This inference appears even more probable since the relationship with dropout at 6 months and breastfeeding history was almost statistically significant, suggesting that women with at least one child are more likely to discontinue a weight-loss intervention.

However, the relationship between lower HDL cholesterol, DBP, and a lower risk of dropout has never been reported elsewhere.

Improving the retention rate of weight-loss programs in this particular population has no simple solutions; however, gaining insight into any predictive factors for dropout at baseline could assist clinicians in devising more personalized approaches to educate these women about modifying their dietary patterns and coping with the potential reasons for discontinuity before they become exacerbated. 

This study represents (to our knowledge), the first attempt to investigate dropout predictors in BCSs following a weight-loss intervention, Nevertheless, some limitations should be noted for our study. First, the major limitation is that the follow-up span was too short to highlight any beneficial effect in terms of cancer recurrence and/or mortality, also considering the relatively small sample size. This aspect was also important because it reduced both the power of analyses, especially at T3 and T4. To reduce these specific limitations, we have planned to further collect data on this population, in particular to provide more patients and long-term data on recurrence and survival. Secondly, as previously mentioned, an unmodifiable limitation is the clinical significance of body composition and metabolic improvements, since the results were limited to subjects that completed the follow-ups, while we do not know the outcomes of dropouts. Lastly, there are other potential limitations related to the incompleteness of the collected parameter pool: (i) even if all subjects included were experiencing a sedentary lifestyle, the level of physical activity (PAL) was not recorded; (ii). socio-psychological determinants for quality of life, distress, anxiety, or depression, or specific psychometric testing data, were not measured.

## 5. Conclusions

In conclusion, substantiating the efficacy of dietary intervention comprehensively to prevent cancer relapse necessitates larger, more extended, and more resource-intensive epidemiological investigations. Specifically, these studies are imperative for elucidating the cumulative impact of the Mediterranean Diet (MD) in conjunction with weight loss on long-term cancer recurrence risk and survival. This area lacks sufficient data, underscoring the need for robust research in this domain. In this context, we are planning to provide further data on this population in the next years.

Taking into account the preliminary data indicating younger age as a robust predictor of dropout, it becomes crucial to tailor individualized interventions based on patient age and menopausal status, especially considering that the evidence linking weight loss alone to a decreased risk of breast cancer recurrence in premenopausal women remains inconclusive.

## Figures and Tables

**Table 1:** Baseline demographic, lifestyle, and therapy characteristics of participants (n = 182).

Baseline Characteristics	N (%)
**Age**	
Mean + SD	53.9 ± 10.4
Median (IQR)	53 (15)
**Educational Level (missing = 19)**	
Middle school or lower	66 (40.5%)
High school or technical institutes	73 (44.8%)
Degree (any)	24 (14.7%)
**Menopausal state (missing = 17)**	
Yes	84 (50.9%)
No	81 (49.1%)
**Breastfeeding history (missing = 17)**	
Yes	94 (57%)
No	71 (43%)
**Smoking history (missing = 17)**	
Yes	65 (39.4%)
No	100 (60.6%)
**Habitual alcohol consumption (missing = 18)**	
Yes	66 (40.2%)
No	98 (59.8%)
**Years since first breast cancer diagnosis (missing = 13)**	
From 0 to 4	125 (74%)
5 or more	44 (26%)
**Breast cancer medications (missing = 18)**	
Aromatase inhibitors	62 (37.8%)
SERMs	24 (14.6%)
Anti-HER2	32 (19.5%)
GnRH agonists No therapy Combination of two drugs	4 (2.4%) 20 (12.2%) 22 (13.4%)
*-Aromatase inhibitors + Anti-HER2*	6 (3.7%)
*-Aromatase inhibitors + SERMs*	13 (7.9%)
*-Aromatase inhibitors + GnRH agonists*	1 (0.6%)
*-SERMs + Anti-HER2*	1 (0.6%)
*-SERMs + GnRH agonists*	1 (0.6%)

SERM: selective estrogen receptor modulator; Anti-HER2: human epidermal growth factor receptor 2 blockers; GnRH: gonadotropin-releasing hormone.

**Table 2:** Baseline characteristics of the sample divided into BMI subgroups.

	NW	OW	OB	*p*-Value
**Patients (%)**	**20 (11%)**	**59 (32.4%)**	**103 (56.6%)**	
**Age** (y)	52 (15)	51 (10)	53 (17)	Ns
**BW**(Kg)	61.9 (7.2)	71.2 (7.8)	87.7 (15.8)	<0.001 ^1^
**BMI** (Kg/m2)	23.7 (1.1)	27.6 (2.4)	34.1 (5.5)	<0.001 ^1^
**Waist** (cm)	88.8 ± 6.4	98.5 ± 6.6	112.9 ± 9.8	<0.001 ^1^
**PA (°)**	5.1 ± 0.47	5.2 ± 0.4	5.2 ± 0.5	Ns
**FM%**	29.8 ± 3	34.4 ± 4.5	43.3 ± 4.6	<0.001 ^2^
**ECW %**	50.4 ± 2.6	49.6 ± 2.4	49.7 ± 2.8	Ns
**Glucose** (mg/dL)	85 (11.5)	88(13.5)	96 (17)	<0.001 ^3^
**Insulin** (μUI/mL)	8.7 ± 6.2	11.7 ± 11.6	15.5 ± 9.2	Ns
**HOMA index**	1.79 ± 1.26	2.59 ± 2.5	4.0 ± 3.0	<0.05 ^4^
**Tot Chol** (mg/dL)	204.8 ± 39.4	206.4 ± 35.6	207.9 ± 40.4	Ns
**HDL** (mg/dL)	55 (18)	56.5 (17.5)	50.5 (20)	Ns
**LDL** (mg/dL)	122.8 ± 31.7	124.0 ± 28.6	125.6 ± 39.3	Ns
**Triglycerides** (mg/dL)	91 (69.2)	106 (87)	130.5 (96)	Ns
**SBP** (mmHg)	120 ± 8.9	117.9 ± 15.7	129.1 ± 15.3	<0.01 ^5^
**DBP** (mmHg)	76.2 ± 6.9	72.9 ± 9.4	80.3 ± 11.9	<0.05 ^5^

*p*-values express statistical significance between the subgroups. ^1^ = *p* < 0.001 between each couple; ^2^ = *p* < 0.05 between NW and OW, *p* < 0.001 between NW or OW and OB; ^3^ = *p* < 05 between NW and OB, *p* < 0.01 between OW and OB; ^4^ = *p* < 0.05 between OW and OB; ^5^ = *p* < 0.01 between OW and OB. Data expressed as mean ± standard deviation for normally distributed variables or as median (IQR) for non-normally distributed variables. NW = normal weight; OW= overweight; OB = obese. BW = body weight; BMI = body mass index; Waist = waist circumference; PA = phase angle; FM% = fat mass %; ECW % = extracellular water % of total body water; HOMA = homeostatic model assessment; HDL = high-density lipoprotein; LDL = low-density lipoprotein; SPB = systolic blood pressure; DBP = diastolic blood pressure.

**Table 3:** Baseline descriptive characteristics in completers divided by BMI classes and comparison at 6 months.

	NW		OW		OB	
	T1	T2	T1	T2	T1	T2
**BW** (Kg)	61.9 (3.0)	58.8 (3.7) °°	71.0 (5.4)	66.5 (7.4) °°°	85.2 (16.8)	79.0 (16.0) °°°
**BMI** (Kg/m^2^)	24.0 (1.4)	23.0 (1.6) °°	27.4 (2.6)	26.6 (2.1) °°°	33.5 (4.8)	31.8 (5.7) °°°
**Waist** (cm)	87.6 ± 9.1	84.6 ± 7.2 *	97.9 ± 5.7	94.2 ± 7.6 ***	111.8 ± 8.4	106.9 ± 9.2 ***
**FM %** (% of BW)	28.7 ± 0.5	28.4 ± 1.3	34.1 ± 7.1	33.2 ± 7.2	42.7 ± 4.4	39.4 ± 6.1 *
**ECW %** (% of TBW)	51.1 ± 4.0	52.2 ± 2.2	49.5 ± 2.5	49.2 ± 1.3	48.8 ± 3.3	48.2 ± 5.8
**PA** (°)	5.0 ± 0.7	4.8 ± 0.4	5.2 ± 0.4	5.3 ± 0.2	5.4 ± 0.6	5.6 ± 1.3
**Glucose** (mg/dL)	82.0 (7.0)	83.0 (8.0)	88.5 (14.7)	90.0 (17.7)	96.0 (17.0)	97.0 (20.5)
**Tot Chol** (mg/dL)	222.0 ± 28.3	206.5 ± 6.4	212.9 ± 38.3	207.2 ± 35.7	207.5 ± 39.6	200.5 ± 31.4
**HDL** (mg/dL)	54.0 (5.7)	55.0 (4.8)	57.0 (19.0)	53.0 (24.0)	50.5 (18.5)	52.5 (13.7) °
**Triglycerides** (mg/dL)	77.0 (132.2)	78.5 (68.4)	97.0 (86.0)	95.0 (110.0)	136.5 (94.0)	113.5 (73.5) °

Data expressed as mean ± standard deviation for normally distributed variables or as median (IQR) for non-normally distributed variables. °,°°,°°° = difference between T2-T1 medians evaluated through Wilcoxon Signed-Rank Test. *,*** = difference between T2-T1 means evaluated through *t*-test for paired samples. NW = normal weight; OW = overweight; OB = obese; BW = body weight; BMI = body mass index; Waist = waist circumference; PA = phase angle; FM % = fat mass %; ECW % = extracellular water % of total body water; HDL = high-density lipoprotein.

**Table 4:** Attrition rate during the study time points divided by BMI classes.

	T1	T2	T3
**Time**	**Baseline**	**6 months**	**12 months**
**N (% of the first sample)**	182	101 (55.5)	61 (33.9)
**NW (% of total)**	19 (10.4)	14 (14.5)	9 (24.6)
**OW (% of total)**	57 (31.3)	38 (37.6)	28 (45.9)
**OB (% of total)**	102 (57.3)	49 (47.5)	24 (39.3)
**Class I–II–II obesity (% of OB)**	50.0–30.4–19.6	60.8–21.4–17.8	58.3–25.1–16.6

N = number of patients; NW = normal weight; OW = overweight; OB = obese.

**Table 5:** Baseline data according to dropout at 6 months.

N	Completers 101	Dropouts 77	*p*-Value
**Age (y)**	53.0 (15.0)	52.0 (15.0)	0.528
**Educational** **level**	1 = 40 2 = 41 3 = 13	1 = 25 2 = 29 3 = 11	Χ^2^ = 0.410 *p* = 0.815
**Menopausal** **state**	No = 42 Yes = 53	No = 36 Yes = 30	Χ^2^ = 1.665 *p* = 0.197
**Breastfeeding** **history**	No = 47 Yes = 48	No = 23 Yes = 43	Χ^2^ = 3.390 *p* = 0.066
**Smoking** **history**	No = 58 Yes = 37	No = 39 Yes = 27	Χ^2^ = 0.063 *p* = 0.802
**Habitual alcohol** **consumption**	No = 58 Yes = 36	No = 36 Yes = 30	Χ^2^ = 0.819 *p* = 0.395
**Years since first** **BC diagnosis**	From 0 to 4 = 72 5 or more = 25	From 0 to 4 = 50 5 or more = 18	Χ^2^ = 0.010 *p* = 0.920
**BC** **medications**	Anti-HER2 = 18 Aromatase inhib = 32 SERM = 15 GnRh agonist = 3 Combinations = 14 No therapy = 12	Anti-HER2 = 14 Aromatase inhib = 29 SERM = 9 GnRh agonist = 1 Combinations = 7 No therapy = 6	Χ^2^ = 5.229 *p* = 0.515
**Body weight** (Kg)	78.2 (17.8)	78.3 (18.6)	0.801
**BMI** (Kg/m^2^)	31.4 (6.6)	30.6 (9.5)	0.450
**Waist** (cm)	106.0 ± 11.5	105.5 ± 13.3	0.784
**PA (°)**	5.25 ± 0.46	5.18 ± 0.50	0.548
**FM %**	38.4 ± 7.5	36.8 ± 6.4	0.378
**ECW %**	49.6 ± 2.5	50.0 ± 2.7	0.608
**Glucose** (mg/dL)	90.0 (18.7)	92.0 (14.0)	0.551
**Insulin** (μUI/mL)	13.5 ± 10.3	13.4 ± 7.9	0.943
**HOMA index**	3.28 ± 2.66	3.23 ± 2.14	0.934
**Tot Chol** (mg/dL)	205.3 ± 35.9	211.7 ± 43.4	0.337
**HDL** (mg/dL)	52.5 (17.5)	52.5 (17.5)	0.639
**LDL** (mg/dL)	122.2 ± 32.4	132.2 ± 39.0	0.217
**Triglycerides** (mg/dL)	122.0 (100.0)	123.0 (88.5)	0.863
**SBP** (mmHg)	125.5 ± 14.2	124.6 ± 18.7	0.805
**DBP** (mmHg)	77.7 ± 10.9	78.4 ± 12.0	0.781

Data expressed as mean ± standard deviation for normally distributed variables or as median (IQR) for non-normally distributed variables. Educational level 1: up to middle school, 2: high school or comparable title, 3: any degree; BMI = body mass index; Waist = waist circumference; PA = phase angle; FM % = fat mass %; ECW % = extracellular water % of total body water; HOMA = homeostatic model assessment; HDL = high-density lipoprotein; LDL = low-density lipoprotein; SPB = systolic blood pressure; DBP = diastolic blood pressure.

**Table 6:** Baseline data according to dropout at 12 months.

**N**	**Completers** **61**	**Dropouts** **117**	***p*** **-Value**
**Age (y)**	55.0 (15.0)	51.0 (15.0)	<0.05
**Educational** **level**	1 = 28 2 = 22 3 = 7	1 = 37 2 = 48 3 = 17	Χ^2^ = 2.537 *p* = 0.281
**Menopausal** **state**	No = 20 Yes = 38	No = 58 Yes = 45	Χ^2^ = 7.079 *p* < 0.01
**Breastfeeding** **history**	No = 27 Yes = 31	No = 43 Yes = 60	Χ^2^ = 0.348 *p* = 0.555
**Smoking** **history**	No = 36 Yes = 22	No = 61 Yes = 42	Χ^2^ = 0.125 *p* = 0.723
**Habitual alcohol** **consumption**	No = 38 Yes = 19	No = 56 Yes = 47	Χ^2^ = 2.290 *p* = 0.130
**Years since first** **BC diagnosis**	From 0 to 4 = 42 5 or more = 17	From 0 to 4 = 80 5 or more = 26	Χ^2^ = 0.361 *p* = 0.548
**BC** **medications**	Anti-HER2 = 7 Aromatase inhib = 20 SERM = 8 GnRh agonist = 2 Combinations = 12 No therapy = 8	Anti-HER2 = 25 Aromatase inhib = 41 SERM = 16 GnRh agonist = 2 Combinations = 9 No therapy = 10	Χ^2^ = 9.555 *p* = 0.145
**Body weight** (Kg)	77.5 (18.9)	78.8 (17.8)	0.822
**BMI** (Kg/m^2^)	30.8 (6.7)	30.8 (8.3)	0.578
**Waist** (cm)	106.1 ± 11.9	105.6 ± 12.6	0.773
**PA (°)**	5.14 ± 0.42	5.23 ± 0.50	0.530
**FM%**	34.9 ± 7.1	38.3 ± 6.7	0.113
**ECW %**	50.2 ± 2.5	49.7 ± 2.6	0.528
**Glucose** (mg/dL)	91.0 (19.8)	90.0 (16.0)	0.539
**Insulin** (μUI/mL)	13.9 ± 11.2	13.1 ± 8.4	0.666
**HOMA index**	3.35 ± 2.61	3.20 ± 2.47	0.769
**Tot Chol** (mg/dL)	205.0 ± 37.8	209.5 ± 39.6	0.493
**HDL** (mg/dL)	49.0 (15.5)	55.0 (16.2)	<0.05
**LDL** (mg/dL)	120.9 ± 31.7	130.0 ± 37.5	0.242
**Triglycerides** (mg/dL)	133.0 (105.0)	117.0 (82.0)	0.129
**SBP** (mmHg)	122.3 ± 13.2	126.8 ± 17.1	0.194
**DBP** (mmHg)	74.4 ± 11.3	79.9 ± 10.9	<0.05

Data expressed as mean ± standard deviation for normally distributed variables or as median (IQR) for non-normally distributed variables. Educational level 1: up to middle school, 2: high school or comparable title, 3: any degree; BMI = body mass index; Waist = waist circumference; PA = phase angle; FM% = fat mass %; ECW% = extracellular water % of total body water; HOMA = homeostatic model assessment; HDL = high-density lipoprotein; LDL = low-density lipoprotein; SPB = systolic blood pressure; DBP = diastolic blood pressure.

## Data Availability

The data presented in this study that are not available within the article are available on request from the corresponding author. These data are not publicly available due to privacy policies.
